# Stability of Composite Plates with a Dense System of Ribs in Two Directions

**DOI:** 10.3390/ma19020322

**Published:** 2026-01-13

**Authors:** Jakub Marczak, Martyna Rabenda, Bohdan Michalak

**Affiliations:** 1 Department of Structural Mechanics, Lodz University of Technology, Politechniki 6, 93-590 Łódź, Poland; bohdan.michalak@p.lodz.pl; 2 Department of Concrete Structures, Lodz University of Technology, Politechniki 6, 93-590 Łódź, Poland; martyna.rabenda@p.lodz.pl

**Keywords:** shear stability, composite plate with microstructure, dense system of ribs, tolerance averaging technique, finite element method

## Abstract

**Highlights:**

**What are the main findings?**

- A simple analytical method of stability analysis of a thin plate with a dense system of ribs is derived and verified.
- Issues of uniaxial compression and shear in-plane loadings are investigated.
- All presented results are verified with FEM-based calculations, proving the correctness of the derived solution.

**What are the implications of the main findings?**

- The derived analytical approach allows swift and reliable stability analysis of composites, contrary to FEM-based investigations, where the modelling of microstructure requires a lot of computational resources and long computation times.
- The performed investigations of the influence of material and geometrical properties on the shear stability of the plate allows us to predict critical forces without the necessity of performing time-consuming calculations.

**Abstract:**

This paper presents an easy-to-use analytical method for stability analysis of composite plates with dense bidirectional microstructure. The main characteristic feature of such a defined composite is that due to its periodic nature the obtainable governing partial differential equations are characterised by discontinuous, strongly oscillating coefficients. Such cases bring many difficulties during derivation of their solution. In order to simplify calculations, the initial governing equations are transformed with the use of the tolerance averaging technique, so a system of partial differential equations with constant coefficients is obtained. The most important finding of the presented work is that the form of the mentioned equations is similar to the classic equations, which describe the stability issue of the thin homogeneous plate. Consequently, the analytical solution to such issues is easily obtainable. Moreover, when compared to, for example, finite element method (FEM) analysis, it requires substantially less computation resources, which can be perceived as its superior feature. Therefore, the proposed method is convenient for engineering applications. In this paper, a comparative analysis of the results obtained from the proposed analytical models with the results obtained from the FEM has been carried out. The impact of materials and dimensions of microstructure on the values of critical normal and shear forces has also been analysed.

## 1. Introduction

### 1.1. Research Significance

Non-homogeneous plates have many applications in modern engineering issues, such as aircraft construction, space vehicles and even in traditional civil engineering. The most common ones are sandwich panels. A review of computational methods for such panels can be found in Hu et al. [1]. Another type of composite plates are plates with internal microstructure, which will be analysed in this paper.

There are many papers in the literature considering similar structures, such as stiffened, fibre-reinforced or functionally graded plates. The work of Deng et al. [2] was dedicated to the buckling analysis of unidirectionally stiffened panels. Teter and Kolakowski [3] and Piculin and Može [4] examined the stability of unidirectionally reinforced panels with thin ribs. The distances between the ribs in these works are large enough to effectively use the finite element method for analysis. The stability analysis of fibre-reinforced plates can be found in the works of Taheri and Ebrahimi [5], where the Mori–Tanaka method was used in the homogenization procedure and in the work [6] where a cantilevered multilayer plate of Marco fibre and carbon fibre composites was analysed. Additional issues connected with, e.g., delamination and manufacturing of carbon-fibre-reinforced polymers can be found in [7,8,9]. The literature concerning plates made of functionally graded materials (FGM) is full of various investigations of interesting cases, such as nonlinear vibrations [10,11], thermal buckling [12], dynamic stability [13], dynamics of FGM shells [14] or flexural behaviour of sandwich plates with FGM core [15].

Possible methods to analyse such plates are, among others, the homogenisation technique or finite element method. The latter is a labour-intensive process due to the necessity of detailing the element mesh to the microstructure of the composite. Tolerance averaging technique, presented in this work, is a special modelling procedure similar to homogenisation. It gives the opportunity to replace strongly oscillating discontinuous coefficients of differential equations with constant ones. Consequently, an easy-to-use method for the evaluation of critical forces of the composite is obtainable. Its superior feature is that it requires substantially less computation resources, than, for example, finite element method (FEM) analysis. Thus, the proposed method is convenient for engineering applications.

The aim of the presented work is to obtain and apply a mathematical macroscopic model describing stability of the thin plate with a dense system of ribs in two directions based on the tolerance averaging technique. General rules of modelling within this technique can be found in the literature, cf. Woźniak et al. [16,17]. Applications of this method for the analysis of various dynamic and thermomechanical problems can be found in multiple works. In the paper presented by Matysiak and Nagórko [18], propagation of harmonic waves in a periodically laminated composite is analysed. The analysis of dynamic behaviour of honeycomb-based composite solids can be performed according to the work of Wierzbicki and Woźniak [19]. In the paper [20], modelling of the dynamics of thin plates made of functionally graded material (FGM) is considered. The obtained averaged equations of motion are verified by comparing the results from the tolerance model with those from the finite element method. The dynamics of three-layer plates (sandwich plates) is analysed in the work of Marczak and Jędrysiak [21], while Domagalski et al. [22] analyse the vibrations of a periodic Timoshenko beam with nonlinearity of the von Karman type. Jędrysiak and Ostrowski [23] describe natural vibrations of a plate made of material of uncertain properties. The description of thermomechanical issues may be found in [24]. Ostrowski and Michalak [25] study an FGM conductor, where the heat conduction in a microheterogeneous cylinder is verified using the finite element method.

The application of the tolerance averaging technique to model stability issues of various periodic composites can be found in a series of articles. The stability of lightly wrinkled plates is analysed by Michalak [26]. Stability of thin periodically stiffened cylindrical shells is investigated in the work [27]. Marczak and Jędrysiak [28] study the stability of periodic beams interacting with elastic foundation, while the vibration and stability of viscoelastic periodic beams based on a periodic foundation with damping is considered in [29]. What is more, the application of the tolerance averaging technique to obtain a macroscopic model describing the stability of plates made of FGM is shown in [30] and is used in the paper [31], where the stability issue of a functionally variable annular plate cooperating with an elastic microheterogenous subsoil is described.

It should be noted that in the abovementioned works the cell dimension has to be much larger than the thickness of the structure. The fundamental distinctive feature of the presented work is that the considered plate is reinforced by a bidirectional rib system, which microstructure length parameter *l* has a dimension similar to the plate thickness *h* (h≅l).

### 1.2. Purpose and Scope of Work

**Figure 1:**
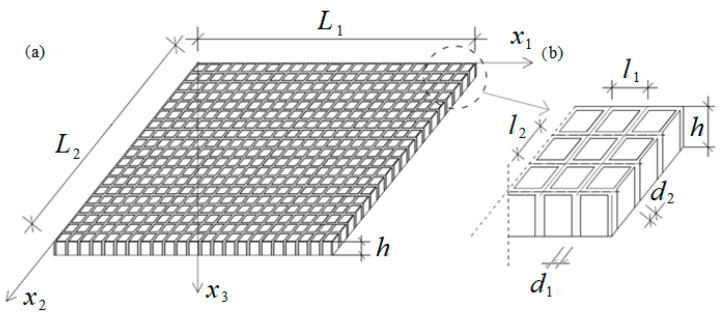
The considered thin plate with a dense system of ribs: (**a**) overview of the composite, (**b**) zoom of a small part with denotations used to describe certain periodic microstructure.

In this work, composite plates with a dense system of ribs are analysed, cf. Figure 1. The main characteristic feature of this contribution is that its periodicity cell dimension of the microstructure is comparable to the thickness of the plate. The main purpose of this work is to obtain and apply a macroscopic mathematical model describing the stability of the plate under normal and shear loadings. A tolerance-averaging technique is used to build the model, which does not contain equations with discontinuous and strongly oscillating coefficients. Eventually, the results obtained from the tolerance-averaging technique are compared with the ones derived from finite element method (FEM). The analysis of the impact of materials and dimensions on critical shear forces is described as well.

## 2. Methods of Calculation

### 2.1. Description of the Considered Composite

Let us analyse a thin plate in a Cartesian coordinate system $0x_{1}x_{2}x_{3}$ with characteristic dimensions $L_{1}$ and $L_{2}$ along the $x_{1}$ and $x_{2}$ axis, respectively, cf. Figure 1. The considered object is a thin composite with constant thickness *h* and a dense bidirectional system of *ribs*. Throughout the article, *ribs* should be understood as those parts of the thin plate which are made of material having superior properties, when compared to the rest of the composite (the so-called *matrix*).

Let us assume that it is possible to distinguish two families of thin homogeneous ribs with widths $d_{1}$ and $d_{2}$, respectively, which axes intersect at right a angle, cf. Figure 1. The areas between the ribs are filled with homogeneous matrix material. Additionally, let us state that it is possible to distinguish *m* ribs along the $x_{1}$ axis and n ribs along the $x_{2}$ axis in the whole structure, where $\frac{1}{n}\ll 1,\;\frac{1}{m}\ll 1.$ Those ribs have a constant width within a specific direction and are distributed uniformly throughout the whole plate. The microstructure parameter $l=\sqrt{l_{1}l_{2}}$ (*l*_1_ and *l*_2_ are periodicity cell dimensions) is assumed to be sufficiently small when compared to the dimensions of the plate midplane, but at the same time it is comparable to the plate thickness *h* (h≅l). A macroscopic model of dynamics of such a type of plate can be found in the work of Michalak and Rabenda [32].

Let us introduce the concept of a basic periodicity cell; $∆\equiv \left(-\frac{l_{1}}{2},\frac{l_{1}}{2}\right)\times \left(-\frac{l_{2}}{2},\frac{l_{2}}{2}\right)$ as the smallest repeatable part of the plate, where $l_{1}=\frac{L_{1}}{m}$ and $l_{2}=\frac{L_{2}}{n}$ are the cell’s dimensions. Moreover, it is assumed that the whole plate is made of isotropic materials with a constant value of Poisson’s ratio. Setting **x** ≡ **(***x*_1_, *x*_2_**)** and $z\equiv x_{3}$, the undeformed plate occupies the region $\Omega \equiv \left\{\left(x,\;z\right):\;-\frac{h}{2}\leq z\leq \frac{h}{2},\;x\in \Pi \right\}$, where Π is the plate’s midplane and h is the constant plate’s thickness.

### 2.2. Formulation of the Preliminary Governing Equations

Let us start with the direct description of the considered plate in the framework of the well-known second order non-linear theory of thin plates. The displacement field of the arbitrary point of the plate is assumed in the form

(1) $$\begin{array}{c} w_{3}\left(x,z\right)=w_{3}\left(x\right),\\ w_{\alpha }\left(x,z\right)=w_{\alpha }^{0}\left(x\right)-z\cdot \partial_{\alpha }w_{3}\left(x\right), \end{array}$$

where $w_{i}\left(x,z\right)$, i=1,2,3, are displacements along the $x_{i}$-axis directions and $w_{\alpha }^{0}\left(x\right)$, α=1,2, are in-plane displacements caused by in-plane loadings.

Denoting by $g_{\alpha \beta }$ the metric tensor, the Ricci tensor by $\epsilon_{\alpha \beta }$, setting $\partial_{k}=\frac{\partial }{\partial x_{k}}$ and the gradient operators $\nabla \equiv \left(\partial_{1},\;\partial_{2}\right)$, where $\nabla_{\alpha }$ and $\nabla_{\alpha \beta }$ are first- and second-order gradients, a system of governing equations of linear approximated theory for thin plates (cf. [33]) is obtained:

(i) strain-displacement relations:

(2) $$\begin{array}{c} e_{\alpha \beta }\left(x,z\right)=\varepsilon_{\alpha \beta }\left(x\right)+\kappa_{\alpha \beta }\left(x\right)\cdot z,\\ \varepsilon_{\alpha \beta }=\nabla_{\beta }w_{\alpha }^{0}+\frac{1}{2}\partial_{\alpha }w_{3}\partial_{\beta }w_{3},\\ \kappa_{\alpha \beta }=-\nabla_{\alpha \beta }w_{3}, \end{array}$$

(ii) strain energy averaged over the plate thickness:

(3) $$E\left(x\right)=\frac{1}{2}B^{\alpha \beta \gamma \delta }\kappa_{\alpha \beta }\kappa_{\gamma \delta }+\frac{1}{2}D^{\alpha \beta \gamma \delta }\varepsilon_{\alpha \beta }\varepsilon_{\gamma \delta },$$

where $D^{\alpha \beta \gamma \eta }=\frac{Eh}{\left(1-\nu^{2}\right)}H^{\alpha \beta \gamma \eta }$ is the tensile stiffness and $B^{\alpha \beta \gamma \eta }=\frac{Eh^{3}}{12\left(1-\nu^{2}\right)}H^{\alpha \beta \gamma \eta }$ with $H^{\alpha \beta \gamma \eta }=0.5\cdot \left(g^{\alpha \eta }g^{\beta \gamma }+g^{\alpha \gamma }g^{\beta \eta }+\upsilon \left(\epsilon^{\alpha \gamma }\epsilon^{\beta \eta }+\epsilon^{\alpha \eta }\epsilon^{\beta \gamma }\right)\right)$ is the bending stiffness.

(iii) work of external forces:

(4) $$F=p^{\alpha }w_{\alpha }^{0}+{p^{3}w}_{3},$$

where $p^{i}$, i=1,2,3, are external loadings applied to the midplane of the considered composite along the $x_{i}$-axis, respectively.

In order to derive governing equations of the considered plate we shall define the stationary action functional:

(5) $$A\left(w\left(\cdot \right)\right)=\int_{\Pi }L\left(w,\;\nabla w,\;\nabla^{2}w\right)dx,$$

where Lagrangean L=F−E. From stationary action principle (δA=0) we obtain governing equations:

(6) $$\begin{array}{c} \partial_{\alpha \beta }m^{\alpha \beta }-\partial_{\alpha }\left(n^{\alpha \beta }\partial_{\alpha }w_{3}\right)=p^{3},\\ \partial_{\beta }n^{\alpha \beta }=-p^{\alpha }, \end{array}$$

where generalised forces are defined as:

(7) $$\begin{array}{c} n^{\alpha \beta }=\int_{-h/2}^{h/2}\sigma^{\alpha \beta }dz=D^{\alpha \beta \gamma \delta }e_{\gamma \delta },\\ m^{\alpha \beta }=\int_{-h/2}^{h/2}\sigma^{\alpha \beta }zdz=B^{\alpha \beta \gamma \delta }\kappa_{\gamma \delta }. \end{array}$$

It can be observed that this direct description leads to a system of partial differential equations with discontinuous and highly oscillating coefficients, which are difficult to use in multiple issues of modern engineering. The above equations are used as a preliminary formulation for the tolerance averaging modelling procedure.

### 2.3. Basic of the Tolerance Modelling

In order to derive averaged equations for the composite plate under consideration we applied the tolerance averaging approach [16,17]. This technique is based on the concept of indiscernibility (cf. [34,35]) and tolerance relations. Let us mention here some basic concepts of this approach, such as an averaging operator, a tolerance periodic function, a slowly varying function and a highly oscillating function.

The fundamental concept of the modelling technique is *the averaging operation*:

(8) $$\langle f\rangle \left(x\right)=\frac{1}{\left|\left.∆\right|\right.}\int_{∆\left(x\right)}f\left(y,\;x\right)dy,\;x\epsilon \bar{\Pi }.$$

We shall refer to Formula (8) as an averaging of arbitrary integrable function $f\left(\cdot \right)$ for every $x\epsilon \bar{\Pi }$.

The main concept of the tolerance averaging approach is that values of functions defined on a region Π can be determined only with a certain accuracy δ. Let δ stand for an arbitrary positive number and let X be a linear normed space. Tolerance relation ≈ for a certain δ is defined by:

(9) $$\left(\forall \left(x_{1},\;x_{2}\right)\in X^{2}\right)\left[x_{1}\approx x_{2}\Leftrightarrow {\left\|x_{1}-x_{2}\right\|}_{x}\leq \delta \right],$$

where δ is called the tolerance parameter. Such a relation can be treated as the already mentioned indiscernibility relation between $x_{1}$ and $x_{2}$.

*Periodic approximation*. Let $H^{r}$ be the Sobolev space for fixed r≥0. Function ${\tilde{f}}^{\left(k\right)}\left(x,\;\cdot \right)\in H^{0}\left(\Pi \right)$, x∈Π, k=1, 2, …, r will be referred to as the periodic approximation of $\partial^{k}f\left(\cdot \right)$ in $∆\left(x\right)$ (where $\partial^{k}$-k-th gradient in Π). For k=0 we define $\partial^{0}f\equiv f$, ${\tilde{f}}^{\left(0\right)}\equiv \tilde{f}$.

*Tolerance periodic function*. Function $f\in H^{r}\left(\Pi \right)$ is called the tolerance periodic function (with respect to cell $∆\left(x\right)$ and tolerance parameter δ), $f\in {TP}_{\delta }^{r}\left(\Pi ,∆\right)$, if for k=1,2,…,r the following conditions are met:

(10) $$\begin{array}{c} \left(\forall x\in \Pi \right)\left(\exists {\tilde{f}}^{\left(k\right)}\left(x,\;\cdot \right)\in H^{0}\left(∆\right)\right)[{||{\partial^{k}f\left(\cdot \right)|}_{\Pi_{x}}-{\tilde{f}}^{\left(k\right)}\left(x,\;\cdot \right)||}_{H^{0}\left(\Pi_{x}\right)}\leq \delta ],\\ \int_{∆\left(\cdot \right)}{\tilde{f}}^{\left(k\right)}\left(\cdot ,\;y\right)dy\in C^{0}\left(\bar{\Pi }\right). \end{array}$$

*Slowly varying function*. Function $F\in H^{r}\left(\Pi \right)$ will be called *the slowly varying function* (with respect to the cell $∆\left(x\right)$ and tolerance parameter δ), and denoted by $F\in {SV}_{\delta }^{r}\left(\Pi ,\;∆\right)$, if for k=1, 2, …, r the following conditions are met:

(11) $$\begin{array}{c} F\in {TP}_{\delta }^{r}\left(\Pi ,\;∆\right),\\ \left(\forall x\in \Pi \right)\left({\tilde{F}}^{\left(k\right)}{\left(x,\;\cdot \right)|}_{∆\left(x\right)}=\partial^{k}F\left(x\right)\right). \end{array}$$

It can be observed that periodic approximation ${\tilde{F}}^{\left(k\right)}\left(x,\;\cdot \right)$ of $\partial^{k}F\left(x\right)$ in $\Delta \left(x\right)$ is a constant function for every x∈Π. In other words, if $F\in {SV}_{\delta }^{r}\left(\Pi ,\;∆\right)$, then:

(12) $$\left(\forall x\in \Pi \right)\left({\left\|\partial^{k}F\left(\cdot \right)-\partial^{k}F\left(x\right)\right\|}_{H^{0}\left(∆\left(x\right)\right)}\leq \delta ,\;k=0,1,\ldots ,r\right).$$

*Highly oscillating function*. Function $\phi \in H^{r}\left(\Pi \right)$ is called *the highly oscillating function* (with respect to the cell $∆\left(x\right)$ and tolerance parameter δ), and denoted by $\phi \in HO_{\delta }^{r}\left(\Pi ,\Delta \right)$, if for k=1, 2, …, r the following conditions are held:

(13) $$\begin{array}{c} \phi \in TP_{\delta \varepsilon }^{r}\left(\Pi ,\Delta \right),\\ \left(\forall x\in \Pi \right)\left[{\left.\left[{\tilde{\phi }}^{\left(k\right)}\left(x,\cdot \right)\right]\right|}_{∆\left(x\right)}=\partial^{k}\tilde{\phi }\left(x,\cdot \right)\right], \end{array}$$

which leads to the following conclusion:

$$\forall F\in SV_{\delta }^{r}\left(\Pi ,\Delta \right)\left(f\equiv \phi F\in TP_{\delta }^{r}\left(\Pi ,\Delta \right)\right)\wedge {\left.{\tilde{f}}^{\left(k\right)}\left(x,\cdot \right)\right|}_{∆\left(x\right)}={\left.F\left(x\right)\partial^{k}\tilde{\phi }\left(x\right)\right|}_{∆\left(x\right)}.$$

Let us by $\varphi \left(\cdot \right)$ denote a highly oscillating function, $\varphi \in HO_{\varepsilon }^{2}\left(\Pi ,\Delta \right)$, defined on $\bar{\Pi }$, continuous together with gradient $\partial^{1}\varphi$. Its second derivative, $\partial^{2}\varphi ,$ is piecewise continuous and bounded. Function $\varphi \left(\cdot \right)$ is called the fluctuation shape function of the second kind, if it depends on l as a parameter and satisfies conditions:

(14) $$\begin{array}{c} 1{}^{\circ}\;\partial^{k}\varphi \in O\left(l^{\alpha -k}\right)\mathrm{for}\;k=1,2,\ldots ,\alpha ,\alpha =2,\\ 2{}^{\circ}\;\langle \varphi \rangle \left(x\right)\approx 0\mathrm{for}\;x\in \Pi_{\Delta }. \end{array}$$

The set of all fluctuation shape functions of the second kind is denoted by $FS_{\delta }^{2}\left(\Pi ,\Delta \right)$.

### 2.4. Tolerance Averaging Technique in the Modelling of Microperiodic Plate

In this section the averaged equations of thin plate are presented. Since the derivation of such equations require a specific use of the tolerance averaging technique, those investigations can be treated as an original content of this work.

The starting point for the derivation of the averaged governing equations for the plate under consideration are Equations (1)–(7). The modelling technique is based on the tolerance averaging approximation and on the micro-macro decomposition of the displacement fields given by:

(15) $$\begin{array}{c} w_{3}\left(x,z\right)=V_{3}\left(x\right),\\ w_{\alpha }\left(x,z\right)=w_{\alpha }^{0}\left(x\right)-z\cdot \partial_{\alpha }w_{3}\left(x\right)=V_{\alpha }\left(x\right)+{\tilde{h}}^{A}\left(y,x\right)V_{\alpha }^{A}\left(x\right)+z\cdot \left(-\partial_{\alpha }V_{3}\left(x\right)+{\tilde{h}}^{A}\left(y,x\right)u_{\alpha }^{A}\left(x\right)\right). \end{array}$$

for x∈Π, $z\in \left(-h/2,h/2\right)$ and A=1,2. The basic tolerance modelling assumption states that macro-displacements $V_{3}\left(\cdot \right),V_{\alpha }\left(\cdot \right)$ and amplitudes of displacements fluctuation $V_{\alpha }^{A}\left(\cdot \right),u_{\alpha }^{A}\left(\cdot \right)$ are slowly varying functions together with all partial derivatives. Functions $V_{3}\left(\cdot \right)\in SV_{\varepsilon }^{2}\left(\Pi ,\Delta \right),V_{\alpha }\left(\cdot \right)\in SV_{\varepsilon }^{1}\left(\Pi ,\Delta \right),V_{\alpha }^{A}\left(\cdot \right)\in SV_{\varepsilon }^{1}\left(\Pi ,\Delta \right),u_{\alpha }^{A}\left(\cdot \right)\in SV_{\varepsilon }^{1}\left(\Pi ,\Delta \right)$ are the basic unknowns. Functions ${\tilde{h}}^{A}\left(\cdot \right)$ are known fluctuation shape functions, which are dependent on microstructure length parameter l. These functions describe the shape of displacements fluctuations in cell $∆\left(x\right)$ caused by the microstructure of the plate.

Let $h^{A}\left(\cdot \right),{\partial_{\alpha }h}^{A}\left(\cdot \right)$ be a periodic approximation of ${\tilde{h}}^{A}\left(\cdot \right),{\partial_{\alpha }\tilde{h}}^{A}\left(\cdot \right)$ in cell $∆\left(x\right)$, respectively. Due to the fact that $w_{3}\left(\cdot \right),w_{\alpha }\left(\cdot \right)$ are tolerance-periodic functions, it can be stated that the periodic approximation of $w_{3h}\left(\cdot ,x\right),w_{\alpha h}\left(\cdot ,x\right)$ and their derivatives in $∆\left(x\right)$, x∈Π can be defined as:

(16) $$\begin{array}{c} w_{3h}\left(y,\;x\right)=V_{3}\left(x\right),\\ \partial_{\alpha }w_{3h}\left(y,x\right)=\partial_{\alpha }V_{3}\left(x\right),\\ w_{\alpha h}\left(y,x,z\right)=V_{\alpha }\left(x\right)+h^{A}\left(y,\;x\right){\;V}_{\alpha }^{A}\left(x\right)+\left(h^{A}\left(y,x\right){\;u}_{\alpha }^{A}\left(x\right)-\partial_{\alpha }V_{3}\left(x\right)\right)z,\\ \partial_{\gamma }w_{\alpha h}\left(y,\;x,z\right)=\partial_{\gamma }V_{\alpha }\left(x\right)+{\partial_{\gamma }h}^{A}\left(y,\;x\right){\;V}_{\alpha }^{A}\left(x\right)+\left(\partial_{\gamma }h^{A}\left(y,\;x\right){\;u}_{\alpha }^{A}\left(x\right)-\partial_{\alpha \gamma }V_{3}\left(x\right)\right)z, \end{array}$$

for every x∈Π and almost every $y\in ∆\left(x\right)$.

By setting $w_{3}=w_{3h}$ and $w_{\alpha }=w_{\alpha h}$ into Lagrangian $\tilde{L}\left(w,\nabla w,\;\nabla^{2}w\right)$ we can assume that $\tilde{L_{h}}\left(w_{h},\nabla w_{h},\;\nabla^{2}w_{h}\right)\in {HO}_{\varepsilon }^{0}\left(\Pi ,\Delta \right)$. Hence the periodic approximation of $\tilde{L_{h}}\left(\cdot \right)$ in every $∆\left(x\right)$ we denote by $L_{h}\left(x,\;y{,w}_{3h},w_{\alpha h},\partial_{\alpha }w_{3h},\partial_{\alpha }w_{\beta h}\right)$. In order to derive the governing equations we shall define tolerance-averaged Lagrangian $\langle L_{h}\rangle =\langle F_{h}\rangle -\langle E_{h}\rangle$:

(17) $$\langle L_{h}\rangle \left(x,\;\nabla_{\alpha \beta }V_{3},\;\nabla_{\alpha }V_{\beta },\;\nabla_{\alpha }V_{3},\;V_{3,\;\;}V_{\alpha },V_{\alpha }^{A},u_{\alpha }^{A}\right)=\frac{1}{\left|∆\right|}\int_{∆\left(x\right)}L_{h}\left(x,\;y,\;w_{3h},\;{w_{\alpha h},\;\partial }_{\alpha }w_{3h},\;\partial_{\alpha }V_{\beta h}\right)dy.$$

Substituting the right-hand sides of Equation (15) into (3), with use of the tolerance averaging technique, we arrive at the following formula for the strain energy averaged over the cell $∆\left(x\right)$:

(18) $$\begin{array}{c} \langle E_{h}\rangle =\frac{1}{2}\langle B^{\alpha \beta \gamma \delta }\rangle \nabla_{\alpha \beta }V_{3}\nabla_{\gamma \delta }V_{3}-\langle B^{\alpha \beta \gamma \delta }\partial_{\gamma }h^{A}\rangle u_{\delta }^{A}\nabla_{\alpha \beta }V_{3}+\frac{1}{2}\langle B^{\alpha \beta \gamma \delta }\partial_{\beta }h^{A}\partial_{\delta }h^{B}\rangle u_{\alpha }^{A}u_{\gamma }^{B}\\ +\frac{1}{2}\langle D^{\alpha \beta \gamma \delta }\rangle \nabla_{\beta }V_{\alpha }\nabla_{\delta }V_{\gamma }+\langle D^{\alpha \beta \gamma \delta }\partial_{\gamma }h^{A}\rangle {V_{\delta }^{A}\nabla }_{\beta }V_{\alpha }+\frac{1}{2}\langle D^{\alpha \beta \gamma \delta }\partial_{\beta }h^{A}\partial_{\delta }h^{B}\rangle V_{\alpha }^{A}V_{\gamma }^{B}\\ +\frac{1}{2}\langle D^{\alpha \beta \gamma \delta }\rangle \nabla_{\beta }V_{\alpha }\nabla_{\gamma }V_{3}\nabla_{\delta }V_{3}+\frac{1}{2}\langle D^{\alpha \beta \gamma \delta }\partial_{\beta }h^{A}\rangle V_{\alpha }^{A}\nabla_{\gamma }V_{3}\nabla_{\delta }V_{3}\\ +\frac{1}{2}\langle D^{\alpha \beta \gamma \delta }\rangle \nabla_{\alpha }V_{3}\nabla_{\beta }V_{3}\nabla_{\gamma }V_{3}\nabla_{\delta }V_{3}. \end{array}$$

The averaged energy of the external loadings over the cell $∆\left(x\right)$ is defined as:

(19) $$\langle F_{h}\rangle =\langle p^{3}\rangle V_{3}+\langle p^{\alpha }\rangle V_{\alpha }+\langle p^{\alpha }h^{A}\rangle V_{\alpha }^{A}.$$

From the principle of stationary action of the averaged Lagrangean $\langle L_{h}\rangle$ we derive equations, which describe

(i) plane stress state:

(20) $$\begin{array}{c} \nabla_{\beta }N^{\alpha \beta }+\langle p^{\alpha }\rangle =0,\\ \langle n^{\alpha \beta }\nabla_{\beta }h^{A}\rangle -\langle p^{\alpha }h^{A}\rangle =0, \end{array}$$

where averaged normal forces are equal to

(21) $$N^{\alpha \beta }=\langle n^{\alpha \beta }\rangle =\langle D^{\alpha \beta \gamma \delta }\rangle \nabla_{\delta }V_{\gamma }+\langle D^{\alpha \beta \gamma \delta }\nabla_{\delta }h^{A}\rangle V_{\gamma }^{A}+\frac{1}{2}\langle D^{\alpha \beta \gamma \delta }\rangle \nabla_{\gamma }V_{3}\nabla_{\delta }V_{3}.$$

(ii) bending state:

(22a) $$\nabla_{\alpha \beta }\left({\tilde{B}}^{\alpha \beta \gamma \delta }\nabla_{\gamma \delta }V_{3}-{\tilde{B}}^{\gamma A\alpha \beta }u_{\gamma }^{A}\right)-\nabla_{\alpha }\left({{N^{\alpha \beta }\nabla }_{\beta }V}_{3}\right)-\langle p^{3}\rangle =0,$$

(22b) $${{\tilde{B}}^{\alpha A\gamma \delta }\nabla }_{\gamma \delta }V_{3}-{\tilde{B}}^{\alpha A\gamma B}u_{\gamma }^{B}=0,$$

where

$${\tilde{B}}^{\alpha \beta \gamma \delta }=\langle B^{\alpha \beta \gamma \delta }\rangle ,\;\;\;\;{\tilde{B}}^{\alpha A\gamma \delta }=\langle B^{\alpha \beta \gamma \delta }\nabla_{\beta }h^{A}\rangle ,\;\;\;\;\;\;{\tilde{B}}^{\alpha A\gamma B}=\langle B^{\alpha \beta \gamma \delta }\nabla_{\beta }h^{A}\nabla_{\delta }h^{B}\rangle .$$

We can observe that from Equation (22b), direct representation of oscillation amplitudes $u_{\gamma }^{B}$ can be obtained. Let $K_{\alpha \beta }^{AB}$ stand for a linear transformation operator such that $K_{\alpha \tau }^{AC}{\tilde{B}}^{\alpha A\gamma B}=\delta_{\tau \gamma }\delta^{BC}$. Thus amplitudes of oscillation are defined as

(23) $$u_{\mu }^{B}=K_{\mu \alpha }^{BA}{\tilde{B}}^{\alpha A\gamma \delta }\nabla_{\gamma \delta }V_{3}.\;\;\;$$

Eventually, by denoting

(24) $$F^{\alpha \beta \gamma \delta }={\tilde{B}}^{\alpha \beta \gamma \delta }-{\tilde{B}}^{\mu B\alpha \beta }K^{\mu B\tau A}{\tilde{B}}^{\tau A\gamma \delta },\;\;$$

we obtain a governing equation for stability of the plate:

(25) $$\nabla_{\alpha \beta }\left(F^{\alpha \beta \gamma \delta }\nabla_{\gamma \delta }V_{3}\right)-\nabla_{\alpha }\left(N^{\alpha \beta }\nabla_{\beta }V_{3}\right)=\langle p^{3}\rangle .\;\;\;\;$$

The above equation has an identical form as the stability equation for thin orthotropic plate. Hence, it is solvable with well-known methods of structural mechanics, which is very useful in practice. Let us once again emphasise that, contrary to classic description, the coefficients in Equation (25) are functional but smooth coefficients.

## 3. Calculation Example and Results

### 3.1. Common Assumptions

In order to verify the correctness of the proposed tolerance model some benchmark analysis should be made. Let us consider a rectangular composite plate with constant width of the ribs and simply supported on all edges.

In the calculation example, the stability of a square plate is investigated under either uniaxial compression or shear stress. In all further calculations it is assumed that the matrix is made of concrete having Young’s modulus $E_{m}=20\;GPa$ and Poisson’s ratio $v_{m}=0.20$, while the ribs are made of steel for which $E_{r}=210\;GPa$ and $v_{r}=0.20$. Additionally, let us state that the considered plate is a biperiodic square plate with characteristic dimensions $L_{1}=L_{2}=4\;m$, thickness h=0.1 m and size of the cell $l_{1}=l_{2}=0.2\;m$. The considered structure is analysed with the use of both presented approaches: tolerance averaging technique and FEM.

### 3.2. Initial Remarks on the Used Tolerance Model

During the whole tolerance modelling procedure, there are several assumptions which have to be made. The important one is the form of fluctuation shape functions. Our further analysis is restricted to the case where it is decided to express the microstructural behaviour of the composite with two shape functions. Both of them are assumed as a certain product, formulated as follows:

(26) $$h^{A}\left(y,\;x\right)=S_{A}\left(y,\;x\right)\cdot \varphi_{A}\left(y,\;x\right),\;\;\;\;$$

where A=I,II.

**Figure 2:**
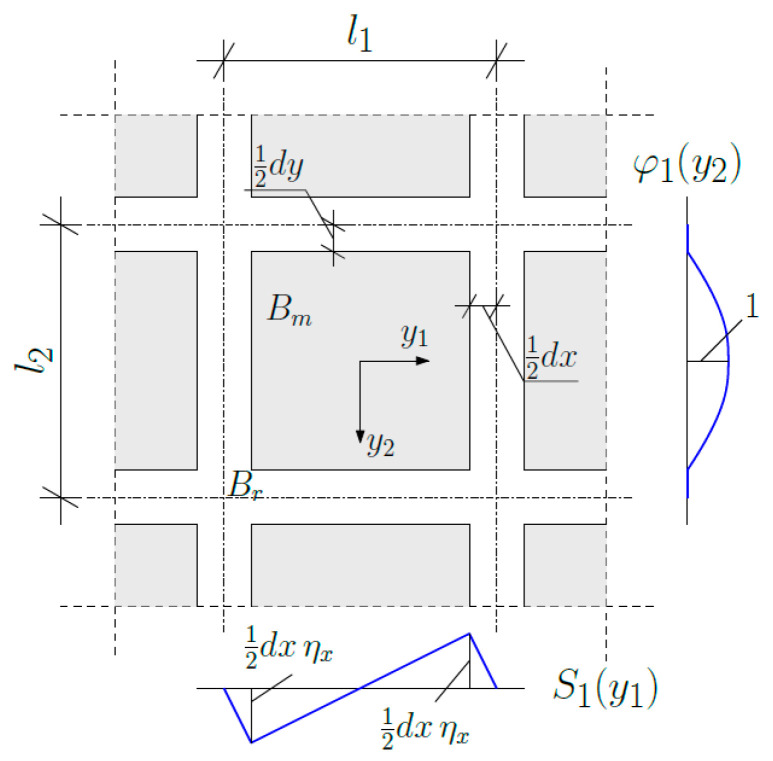
Fluctuation shape function $h^{I}\left(y,\;x\right)$ in the considered cell.

(a) Fluctuation shape function $h^{I}\left(y,\;x\right)$ (cf. Figure 2) is defined as

(27) $$S_{1}\left(y_{1},x_{1}\right)=\left\{\begin{array}{ccc} -\left(y_{1}+\frac{l_{1}}{2}\right)\cdot \eta_{x} & \mathrm{for} & y_{1}\epsilon \langle -\frac{l_{1}}{2},-\frac{b_{1}}{2}\rangle \\ \frac{dx}{l_{1}-dx}\cdot y_{1}\eta_{x} & \mathrm{for} & y_{1}\epsilon \langle -\frac{b_{1}}{2},\frac{b_{1}}{2}\rangle \\ -\left(y_{1}-\frac{l_{1}}{2}\right)\cdot \eta_{x} & \mathrm{for} & y_{1}\epsilon \langle \frac{b_{1}}{2},\frac{l_{1}}{2}\rangle \end{array}\right.,$$

(28) $$\varphi_{1}\left(y_{2},x_{2}\right)=\left\{\begin{array}{ccc} 0 & \mathrm{for}\; & y_{2}\epsilon \langle -\frac{l_{2}}{2},-\frac{b_{2}}{2}\rangle \\ 1-{\left(\frac{2y_{2}}{b_{2}}\right)}^{2} & \mathrm{for}\; & \;y_{2}\epsilon \langle -\frac{b_{2}}{2},\frac{b_{2}}{2}\rangle \\ 0 & \mathrm{for}\; & y_{2}\epsilon \langle \frac{b_{2}}{2},\frac{l_{2}}{2}\rangle \end{array}\right.,$$

where $\eta_{x}=\frac{dx}{l_{1}},\;b_{1}=l_{1}-dx$.

**Figure 3:**
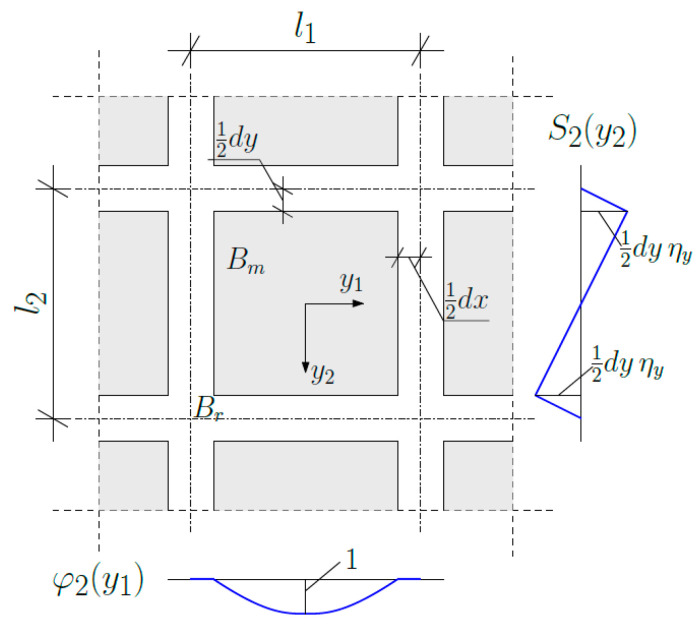
Fluctuation shape function $h^{II}\left(y,\;x\right)\;$ in the considered cell.

(b) Fluctuation shape function $h^{II}\left(y,\;x\right)$ (cf. Figure 3) is defined as

(29) $$S_{2}\left(y_{2},x_{2}\right)=\left\{\begin{array}{ccc} -\left(y_{2}+\frac{l_{2}}{2}\right)\cdot \eta_{y} & \mathrm{for} & y_{2}\epsilon \langle -\frac{l_{2}}{2},-\frac{b_{2}}{2}\rangle \\ \frac{dy}{l_{2}-dy}\cdot y_{2}\eta_{y}\; & \mathrm{for} & y_{2}\epsilon \langle -\frac{b_{2}}{2},\frac{b_{2}}{2}\rangle \\ -\left(y_{2}-\frac{l_{2}}{2}\right)\cdot \eta_{y} & \mathrm{for} & y_{2}\epsilon \langle \frac{b_{2}}{2},\frac{l_{2}}{2}\rangle \end{array}\right.,$$

(30) $$\varphi_{2}\left(y_{1},x_{1}\right)=\left\{\begin{array}{ccc} 0 & \mathrm{for} & y_{1}\epsilon \langle -\frac{l_{1}}{2},-\frac{b_{1}}{2}\rangle \\ 1-{\left(\frac{2y_{1}}{b_{1}}\right)}^{2} & \mathrm{for} & \;y_{1}\epsilon \langle -\frac{b_{1}}{2},\frac{b_{1}}{2}\rangle \\ 0 & \mathrm{for} & y_{1}\epsilon \langle \frac{b_{1}}{2},\frac{l_{1}}{2}\rangle \end{array}\right.,$$

where $\eta_{y}=\frac{dy}{l_{2}},\;b_{2}=l_{2}-dy$.

In the case of composite plate with constant width of the ribs the stability Equation (25) of the tolerance model transforms into

(31) $$\begin{array}{c} F^{1111}\partial_{1111}V_{3}+2\left(F^{1122}+F^{1212}\right)\partial_{1122}V_{3}+F^{2222}\partial_{2222}V_{3}-N^{11}\partial_{11}V_{3}+\\ -{2N}^{12}\partial_{12}V_{3}{-N}^{22}\partial_{22}V_{3}=0, \end{array}$$

where for square plate and $d_{x}=d_{y}=d$ and $l_{1}=l_{2}=l$ we have

(32) $$\begin{array}{c} F^{1111}=F^{2222}={\tilde{B}}^{1111}-\left(1+v^{2}\right){\left({\tilde{B}}^{1I11}\right)}^{2}K^{1I1I}-2v{\left({\tilde{B}}^{1I11}\right)}^{2}K^{1I2II},\\ F^{1122}=v{\tilde{B}}^{1111}-\left(1+v^{2}\right){\left({\tilde{B}}^{1I11}\right)}^{2}K^{1I2II}-2v{\left({\tilde{B}}^{1I11}\right)}^{2}K^{1I1I},\\ F^{1212}=F^{1221}=\frac{1-v}{2}{\tilde{B}}^{1111}-2{\left(\frac{1-v}{2}\right)}^{2}{\left({\tilde{B}}^{1I11}\right)}^{2}\left(K^{1II1II}+K^{1II2I}\right),\\ K^{1I1I}=\frac{{\tilde{B}}^{2II2II}}{{\tilde{B}}^{1I1I}{\tilde{B}}^{2II2II}-{\left({\tilde{B}}^{1I2II}\right)}^{2}}\;,\;K^{1I2II}=\frac{{\tilde{B}}^{1I2II}}{{\tilde{B}}^{1I1I}{\tilde{B}}^{2II2II}-{\left({\tilde{B}}^{1I2II}\right)}^{2}},\\ K^{1II2I}=\frac{-{\tilde{B}}^{II2I}}{{\tilde{B}}^{1I1I}{\tilde{B}}^{2II2II}-{\left({\tilde{B}}^{1I2II}\right)}^{2}}{,\;K}^{1II1II}=\frac{{\tilde{B}}^{2I2I}}{{\tilde{B}}^{2I2I}{\tilde{B}}^{1II1II}-{\left({\tilde{B}}^{1II2I}\right)}^{2}},\\ K^{1I1I}=\frac{{\tilde{B}}^{2II2II}}{{\tilde{B}}^{1I1I}{\tilde{B}}^{2II2II}-{\left({\tilde{B}}^{1I2II}\right)}^{2}}\;,\;K^{1I2II}=\frac{{\tilde{B}}^{1I2II}}{{\tilde{B}}^{1I1I}{\tilde{B}}^{2II2II}-{\left({\tilde{B}}^{1I2II}\right)}^{2}},\\ {\tilde{B}}^{1111}=B_{r}\left[\left(1-n_{d}\right)\left(n_{d}+\alpha \left(1-n_{d}\right)\right)+n_{d}\right],\\ \langle \tilde{\rho }\rangle =\tilde{\rho_{r}}\left[\left(1-n_{d}\right)\left(n_{d}+\beta \left(1-n_{d}\right)\right)+n_{d}\right],\\ {\tilde{B}}^{1I1I}={\tilde{B}}^{2II2II}=B_{r}\left[\frac{8}{15}\left(1-n_{d}+\alpha \cdot n_{d}\right){n_{d}}^{3}+\frac{2\left(1-v\right)}{9}{n_{d}}^{4}\frac{n_{d}+\alpha \left(1-n_{d}\right)}{1-n_{d}}\right],\\ {\tilde{B}}^{1I2II}=\frac{4}{9}B_{r}v\alpha {n_{d}}^{4},\\ {\tilde{B}}^{1II2I}=\frac{2}{9}B_{r}\left(1-v\right)\alpha {n_{d}}^{4},\;\;{\tilde{B}}^{1I11}=\frac{2}{3}B_{r}\left(\alpha -1\right)\left(1-n_{d}\right){n_{d}}^{2},\\ \alpha =\frac{B_{m}}{B_{r}},\;n_{d}=\frac{d}{l}. \end{array}$$

In the above formulas it is assumed that Poisson’s ratio $v=v_{m}=v_{r}$ and $B_{m}$, $B_{r}$ as the stiffness of the matrix and ribs, respectively. In such form, the derived tolerance model of the composite is ready to be used in further calculations.

### 3.3. Finite Element Modelling of the Composite

**Figure 4:**
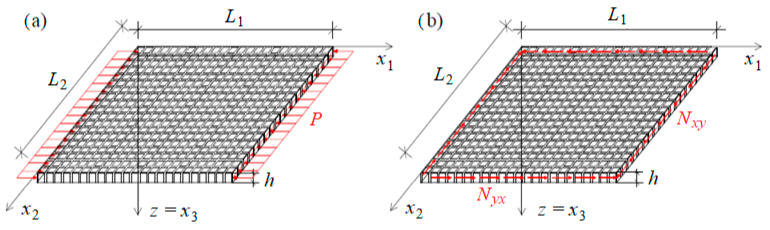
Simply supported plate: (**a**) plate under uniaxial compression, (**b**) plate under shear load.

In order to assess the quality of results derived within the averaged model of a thin plate with a dense system of ribs, the FEM formulation of the same structure is performed, cf. Figure 4. The whole composite is created as a two-dimensional shell plate with a thickness of 0.10 m. Several different geometries are investigated, which differ from each other with the width of ribs $d_{x}$ and $d_{y}$. Obviously, different sets of material properties (elastic moduli and Poisson’s ratios) are applied to both ribs and matrix in every calculation case.

In all calculations it is assumed that the considered plates are rectangular and simply supported on all for edges, which within FEM modelling should be understood as no perpendicular displacements on the whole boundary of the composite. Such a case is ideal for the assessment of quality of the averaged model, as its solution is easily obtainable using mathematical methods.

Let us make several remarks concerning the quality of FEM analysis. As has been stated, the whole structure is modelled with shell elements (S4R and S3) with thickness equal to 0.10 m. Multiple mesh sizes were considered in order to provide reliable results with reasonable computing times. Eventually, all investigated structures were modelled with the use of approximately 100,000–180,000 elements, depending on the ribs’ dimensions. Within such approach the convergence of results is assured.

### 3.4. Calculation Example I—Stability Under Uniaxial Compression

**Figure 5:**
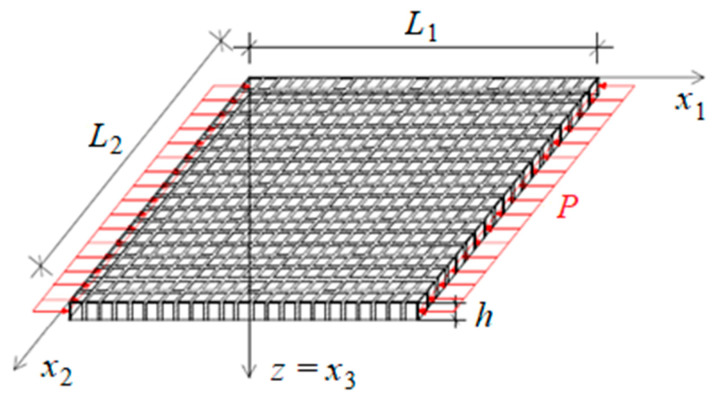
Simply supported plate compressed in the $x_{1}$ direction.

In this section the stability of biperiodic rectangular plate compressed in one direction $\left(x_{1}\right)$ is investigated, cf. Figure 5.

In such a case, the stability Equation (31) is reduced to the form

(33) $$F^{1111}\partial_{1111}V_{3}+2\left(F^{1122}+2F^{1212}\right)\partial_{1122}V_{3}+F^{2222}\partial_{2222}V_{3}+P\partial_{11}V_{3}=0.$$

where $N^{11}=-P$. The above issue is solved in a way which is similar to the known method for a simply supported rectangular homogeneous plate. Let use the following form of solution:

(34) $$V_{3}\left(x\right)=\sum_{m=1}^{\infty }\sum_{n=1}^{\infty }V_{mn}\cdot sin\left(\alpha_{m}x_{1}\right)\cdot sin\left(\beta_{n}x_{2}\right),$$

where $\alpha_{m}=\frac{m\pi }{L_{1}}$, $\beta_{n}=\frac{n\pi }{L_{2}}$ and $V_{mn}$ are coefficients of the sine series corresponding to certain buckling shapes defined by parameters m,n=1,2,3,…. In the next step, after substituting (34) into Equation (33) we obtain

(35) $$P=\frac{\pi^{2}F^{1111}}{L_{1}^{2}}\cdot \frac{\sum_{m=1}^{\infty }\sum_{n=1}^{\infty }\left(m^{4}+\eta \cdot m^{2}n^{2}+n^{4}\right)\cdot V_{mn}}{\sum_{m=1}^{\infty }\sum_{n=1}^{\infty }m^{2}\cdot V_{mn}},$$

where $\eta =2\left(F^{1122}+{2F}^{1212}\right)/F^{1111}$. The critical force for the *m*-th and *n*-th buckling mode is equal to

(36) $$P_{cr}=\frac{\pi^{2}F^{1111}}{L_{1}^{2}}\cdot \frac{m^{4}+\eta \cdot m^{2}n^{2}+n^{4}}{m^{2}}.$$

It is clear that for m=n=1 we obtain the lowest value of the critical force, which is related to the first mode of buckling.

The results of the tolerance model (TM), together with its benchmark results of FEM for the following widths of ribs: $d=\left\{0.00,0.005,0.007,0.01,0.015,0.02,0.025,0.03\right\}m$, are shown in Table 1. We can also notice that the values of critical forces for $n_{d}=0$ are identical to those for a classic homogeneous plate made of concrete with the appropriate value of the elastic modulus.

The results presented in Table 1 require special comment. It can be noticed that the relative error increases with the width of the ribs. It is caused by the growing influence of microheterogeneities in the calculations of critical force. The presented modelling procedure of TM clearly indicates that within this solution the existence of ribs with certain width is averaged on the whole periodicity cell. Such an approach allows us to simplify the initial governing equations so that they are easily solvable.

**Figure 6:**
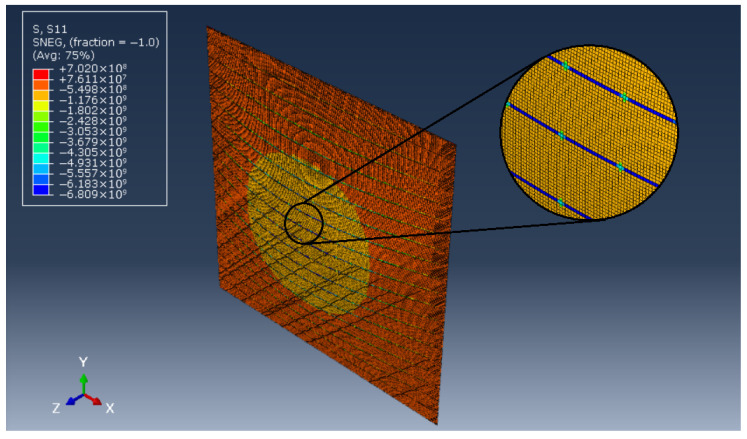
Stress distribution in considered biperiodic plate (d=0.01 m) subjected to compression in the *x* direction, with a zoom of its small part.

On the other hand, these transformations should be always perceived as a kind of approximation, the consequences of which become clear when compared to the explicit modelling of the rib in FEM, cf. Figure 6. In reality, it can be observed that the ribs (made of materials having superior properties when compared to the matrix) carry most of the in-plane loadings. Obviously, the averaging operation in the case of such disproportions must lead to noticeable discrepancies, which become larger with higher widths of ribs. Consequently, the wider the ribs, the lesser precision of the averaged model, which is noticeable in the results presented in Table 1.

Nevertheless, it should be emphasised that for the considered biperiodic plate with a microstructure, the differences in the results between TM and the FEM model are smaller than 10% when $n_{d}\leq 0.05$.

### 3.5. Calculation Example II—Shear Stability

**Figure 7:**
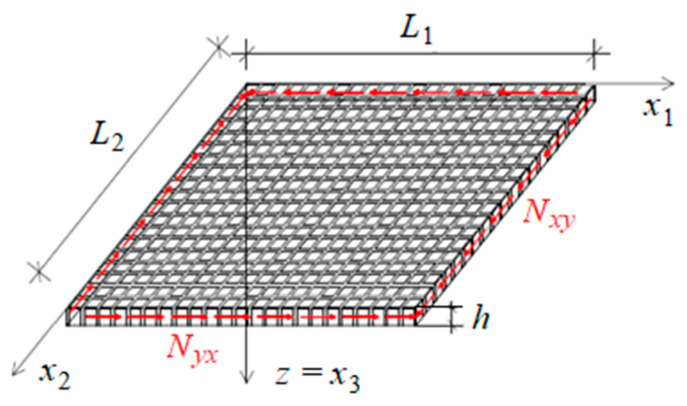
The considered simply supported plate under shear load.

In the current calculations, shear stability of a square plate is analysed. The critical value of the uniform shear load on a simply supported rectangular plate (Figure 7) is determined. Material properties and geometry of the composite are assumed as in Section 3.1. Considering only the strain energy of bending U and the work of the shear load T, the critical value of the load is determined from the condition

(37) U=T.

The strain energy of the considered microstructural plate is equal to

(38) $$U=\frac{1}{2}\iint \left[\left(F^{1111}\frac{\partial^{2}V_{3}}{\partial x^{2}}+F^{1122}\frac{\partial^{2}V_{3}}{\partial y^{2}}\right)\frac{\partial^{2}V_{3}}{\partial x^{2}}+2\left(2F^{1212}\frac{\partial^{2}V_{3}}{\partial x\partial y}\right)\frac{\partial^{2}V_{3}}{\partial x\partial y}+\left(F^{2222}\frac{\partial^{2}V_{3}}{\partial y^{2}}+F^{2211}\frac{\partial^{2}V_{3}}{\partial x^{2}}\right)\frac{\partial^{2}V_{3}}{\partial y^{2}}\right]dA.$$

During the deformation of the plate, the work of the shear load can be expressed as

(39) $$T=-\frac{1}{2}\iint 2N_{xy}\frac{\partial V_{3}}{\partial x}\frac{\partial V_{3}}{\partial y}dA.$$

The solution to the stated issue is assumed in the form

(40) $$V_{3}\left(x\right)=\sum_{m=1}^{\infty }\sum_{n=1}^{\infty }V_{3}^{mn}\cdot sin\left(\alpha_{m}x\right)\cdot sin\left(\beta_{n}y\right),$$

where $\alpha_{m}=m\pi /L_{1}$, $\beta_{n}=n\pi /L_{2}$. Substituting (40) into Formulas (39) and (38), respectively, we obtain

(41) $$U=\frac{L_{1}L_{2}}{8}\sum_{m=1}^{\infty }\sum_{n=1}^{\infty }\left[F^{1111}{\left(\alpha_{m}\right)}^{4}+2\left(F^{1122}+2F^{1212}\right){\left(\alpha_{m}\right)}^{2}{\left(\beta_{n}\right)}^{2}+F^{2222}{\left(\beta_{n}\right)}^{4}\right]{\left(V_{3}^{mn}\right)}^{2},$$

(42) $$T=-4N_{xy}\sum_{m=1}^{\infty }\sum_{n=1}^{\infty }\sum_{p=1}^{\infty }\sum_{q=1}^{\infty }V_{3}^{mn}V_{3}^{pq}\frac{mnpq}{\left(m^{2}-p^{2}\right)\left(q^{2}-n^{2}\right)},$$

where m±p and n±q must be odd numbers. For the even values of m±p or n±q the work of the shear loading T=0, and consequently no buckling modes are obtainable. From the energy equilibrium condition (37) the expression for the critical forces for the *m*-th and *n*-th buckling mode is obtained:

(43) $$N_{xy,cr}=-\frac{L_{1}L_{2}}{32}\frac{\sum_{m=1}^{\infty }\sum_{n=1}^{\infty }\left[F^{1111}{\left(\alpha_{m}\right)}^{4}+2\left(F^{1122}+2F^{1212}\right){\left(\alpha_{m}\right)}^{2}{\left(\beta_{n}\right)}^{2}+F^{2222}{\left(\beta_{m}\right)}^{4}\right]{\left(V_{3}^{mn}\right)}^{2}}{\sum_{m=1}^{\infty }\sum_{n=1}^{\infty }\sum_{p=1}^{\infty }\sum_{q=1}^{\infty }\frac{mnpq}{\left(m^{2}-p^{2}\right)\left(q^{2}-n^{2}\right)}}.$$

It can be noticed that it is no longer true that the lowest values of the critical force are obtained for the lowest values of parameters *m*, *p*, *n*, *q*. In such a case, the smallest critical value of the load is obtained by levelling the derivatives of each factor $V_{3}^{mn}$ to zero. Subsequently, a system of linear equations for the unknowns $V_{3}^{mn}$ are received. By limiting the values of the indicators m≤4 and n≤4, this system of equations has the form

(44) $$\left[\begin{array}{cccccccccccccccc} \lambda \alpha_{11} & 0 & 0 & 0 & 0 & -\frac{4}{9} & 0 & -\frac{8}{45} & 0 & 0 & 0 & 0 & 0 & -\frac{8}{45} & 0 & -\frac{16}{225}\\ 0 & \lambda \alpha_{12} & 0 & 0 & \frac{4}{9} & 0 & -\frac{12}{15} & 0 & 0 & 0 & 0 & 0 & \frac{8}{45} & 0 & -\frac{24}{75} & 0\\ 0 & 0 & \lambda \alpha_{13} & 0 & 0 & \frac{12}{15} & 0 & -\frac{24}{21} & 0 & 0 & 0 & 0 & 0 & \frac{24}{75} & 0 & -\frac{48}{105}\\ 0 & 0 & 0 & \lambda \alpha_{14} & \frac{8}{45} & 0 & \frac{24}{21} & 0 & 0 & 0 & 0 & 0 & \frac{16}{225} & 0 & \frac{48}{105} & 0\\ 0 & \frac{4}{9} & 0 & \frac{8}{45} & \lambda \alpha_{21} & 0 & 0 & 0 & 0 & -\frac{12}{15} & 0 & -\frac{24}{75} & 0 & 0 & 0 & 0\\ -\frac{4}{9} & 0 & \frac{12}{15} & 0 & 0 & \lambda \alpha_{22} & 0 & 0 & \frac{12}{15} & 0 & -\frac{36}{25} & 0 & 0 & 0 & 0 & 0\\ 0 & -\frac{12}{15} & 0 & \frac{24}{21} & 0 & 0 & \lambda \alpha_{23} & 0 & 0 & \frac{36}{25} & 0 & -\frac{72}{35} & 0 & 0 & 0 & 0\\ -\frac{8}{45} & 0 & -\frac{24}{21} & 0 & 0 & 0 & 0 & \lambda \alpha_{24} & \frac{24}{75} & 0 & \frac{72}{35} & 0 & 0 & 0 & 0 & 0\\ 0 & 0 & 0 & 0 & 0 & \frac{12}{15} & 0 & \frac{24}{75} & \lambda \alpha_{31} & 0 & 0 & 0 & 0 & -\frac{24}{21} & 0 & -\frac{48}{105}\\ 0 & 0 & 0 & 0 & -\frac{12}{15} & 0 & \frac{36}{25} & 0 & 0 & \lambda \alpha_{32} & 0 & 0 & \frac{24}{21} & 0 & -\frac{72}{35} & 0\\ 0 & 0 & 0 & 0 & 0 & -\frac{36}{25} & 0 & \frac{72}{35} & 0 & 0 & \lambda \alpha_{33} & 0 & 0 & \frac{72}{35} & 0 & -\frac{144}{49}\\ 0 & 0 & 0 & 0 & -\frac{24}{75} & 0 & -\frac{72}{35} & 0 & 0 & 0 & 0 & \lambda \alpha_{34} & \frac{48}{105} & 0 & \frac{144}{49} & 0\\ 0 & \frac{8}{45} & 0 & \frac{16}{225} & 0 & 0 & 0 & 0 & 0 & \frac{24}{21} & 0 & \frac{48}{105} & \lambda \alpha_{41} & 0 & 0 & 0\\ -\frac{8}{45} & 0 & \frac{24}{75} & 0 & 0 & 0 & 0 & 0 & -\frac{24}{21} & 0 & \frac{72}{35} & 0 & 0 & \lambda \alpha_{42} & 0 & 0\\ 0 & -\frac{24}{75} & 0 & \frac{48}{105} & 0 & 0 & 0 & 0 & 0 & -\frac{72}{35} & 0 & \frac{144}{49} & 0 & 0 & \lambda \alpha_{43} & 0\\ -\frac{16}{225} & 0 & -\frac{48}{105} & 0 & 0 & 0 & 0 & 0 & -\frac{48}{105} & 0 & -\frac{144}{49} & 0 & 0 & 0 & 0 & \lambda \alpha_{44} \end{array}\right]\left[\begin{array}{c} V_{3}^{11}\\ V_{3}^{12}\\ V_{3}^{13}\\ V_{3}^{14}\\ V_{3}^{21}\\ V_{3}^{22}\\ V_{3}^{23}\\ V_{3}^{24}\\ V_{3}^{31}\\ V_{3}^{32}\\ V_{3}^{33}\\ V_{3}^{34}\\ V_{3}^{41}\\ V_{3}^{42}\\ V_{3}^{43}\\ V_{3}^{44} \end{array}\right]=0$$

where $\lambda =\frac{L_{1}L_{2}\pi^{4}}{32N_{xy}}$ and

$$\begin{array}{c} \alpha_{11}=\frac{F^{1111}}{L_{1}^{4}}+\frac{2\left(F^{1122}+2F^{1212}\right)}{L_{1}^{2}L_{2}^{2}}+\frac{F^{2222}}{L_{2}^{4}},\\ \alpha_{12}=\frac{F^{1111}}{L_{1}^{4}}+\frac{8\left(F^{1122}+2F^{1212}\right)}{L_{1}^{2}L_{2}^{2}}+\frac{{16F}^{2222}}{L_{2}^{4}},\\ \alpha_{21}=\frac{16F^{1111}}{L_{1}^{4}}+\frac{8\left(F^{1122}+2F^{1212}\right)}{L_{1}^{2}L_{2}^{2}}+\frac{F^{2222}}{L_{2}^{4}},\\ \alpha_{13}=\frac{F^{1111}}{L_{1}^{4}}+\frac{18\left(F^{1122}+2F^{1212}\right)}{L_{1}^{2}L_{2}^{2}}+\frac{{81F}^{2222}}{L_{2}^{4}},\\ \alpha_{31}=\frac{{81F}^{1111}}{L_{1}^{4}}+\frac{18\left(F^{1122}+2F^{1212}\right)}{L_{1}^{2}L_{2}^{2}}+\frac{F^{2222}}{L_{2}^{4}},\\ \alpha_{22}=\frac{{16F}^{1111}}{L_{1}^{4}}+\frac{32\left(F^{1122}+2F^{1212}\right)}{L_{1}^{2}L_{2}^{2}}+\frac{{16F}^{2222}}{L_{2}^{4}},\\ \alpha_{23}=\frac{{16F}^{1111}}{L_{1}^{4}}+\frac{72\left(F^{1122}+2F^{1212}\right)}{L_{1}^{2}L_{2}^{2}}+\frac{{81F}^{2222}}{L_{2}^{4}},\\ \alpha_{32}=\frac{{81F}^{1111}}{L_{1}^{4}}+\frac{72\left(F^{1122}+2F^{1212}\right)}{L_{1}^{2}L_{2}^{2}}+\frac{{16F}^{2222}}{L_{2}^{4}},\\ \alpha_{33}=\frac{{81F}^{1111}}{L_{1}^{4}}+\frac{162\left(F^{1122}+2F^{1212}\right)}{L_{1}^{2}L_{2}^{2}}+\frac{{81F}^{2222}}{L_{2}^{4}},\\ \alpha_{44}=\frac{{256F}^{1111}}{L_{1}^{4}}+\frac{512\left(F^{1122}+2F^{1212}\right)}{L_{1}^{2}L_{2}^{2}}+\frac{{256F}^{2222}}{L_{2}^{4}},\\ \alpha_{14}=\frac{F^{1111}}{L_{1}^{4}}+\frac{32\left(F^{1122}+2F^{1212}\right)}{L_{1}^{2}L_{2}^{2}}+\frac{{256F}^{2222}}{L_{2}^{4}},\\ \alpha_{24}=\frac{{16F}^{1111}}{L_{1}^{4}}+\frac{128\left(F^{1122}+2F^{1212}\right)}{L_{1}^{2}L_{2}^{2}}+\frac{{256F}^{2222}}{L_{2}^{4}},\\ \alpha_{34}=\frac{{81F}^{1111}}{L_{1}^{4}}+\frac{288\left(F^{1122}+2F^{1212}\right)}{L_{1}^{2}L_{2}^{2}}+\frac{{256F}^{2222}}{L_{2}^{4}},\\ \alpha_{41}=\frac{256F^{1111}}{L_{1}^{4}}+\frac{32\left(F^{1122}+2F^{1212}\right)}{L_{1}^{2}L_{2}^{2}}+\frac{F^{2222}}{L_{2}^{4}},\\ \alpha_{42}=\frac{{256F}^{1111}}{L_{1}^{4}}+\frac{128\left(F^{1122}+2F^{1212}\right)}{L_{1}^{2}L_{2}^{2}}+\frac{{16F}^{2222}}{L_{2}^{4}},\\ \alpha_{43}=\frac{{256F}^{1111}}{L_{1}^{4}}+\frac{288\left(F^{1122}+2F^{1212}\right)}{L_{1}^{2}L_{2}^{2}}+\frac{{81F}^{2222}}{L_{2}^{4}}. \end{array}$$

Eventually, a system of eigenvalues of the system of Equation (44) are evaluated, which corresponds to the critical value of the shear force:

(45) $$N_{xy,cr}=\frac{\pi^{4}L_{1}L_{2}}{32\lambda_{max}}.$$

The tolerance model (TM) is verified for the following widths of ribs: $d=\left\{0.00,\;0.005,\;0.007,\;0.01,\;0.015,\;0.02,\;0.025,\;0.03\right\}\;m$. Results of both TM and FEM analysis are shown in Table 2.

Results presented in Table 2 have similar characteristic features as those presented in Table 1. It is clear that the precision of the TM is decreasing in the case of structures with higher disproportions in material properties, which in this calculation are represented by composites with wider ribs. Similarly, as in the case of uniaxial compression, the differences in the results of critical shear forces between TM and the FEM models are smaller than 10% when $n_{d}\leq 0.05$.

### 3.6. Calculation Example III—Influence of Material and Geometrical Properties on Shear Stability of the Plate

In this section, the influence of certain geometry and material properties on the shear stability is investigated. Let us remember that all calculations are performed for a simply supported square plate with dimensions $L_{1}=L_{2}=4.0\;m$ and thickness h=0.1 m. Additionally, all considered cases are characterised by ribs made of steel $\left(E_{r}=210\;GPa,\;\nu_{r}=0.2\right)$.

**Figure 8:**
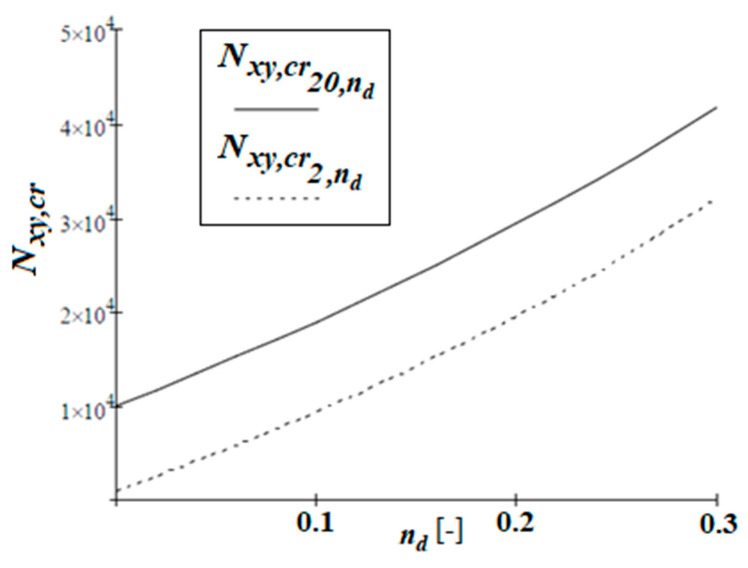
Critical forces $N_{{xy,cr}_{20,nd}}$ and $N_{{xy,cr}_{2,nd}}$ for a matrix made of concrete with Young’s modulus $E_{m20}=20\;GPa$ and $E_{m2}=2\;GPa$ versus ratio $n_{d}\epsilon \langle 0.0;0.30\rangle ,$ respectively.

Firstly, the influence of the coefficient $n_{d}=d/l$ (*d*—rib’s width, *l*—cell’s dimension) on the critical value of shear forces is evaluated. The range of the assumed ratio is assumed as $n_{d}\epsilon \langle 0.0;0.3\rangle$. The calculations are made for two different matrix materials: typical concrete with Young’s modulus of $E_{m20}=20\;GPa$ and light concrete with $E_{m2}=2\;GPa$. Figure 8 shows the critical values of shear forces versus ratio $n_{d}$.

The nature of the graphs in Figure 8 is almost linear (slightly exponential). It can be seen that the ratio $\frac{N_{{xy,cr}_{2,nd}}}{N_{{xy,cr}_{20,nd}}}$ increases as the parameter $n_{d}$ increases. On the other hand, the difference between the values $N_{{xy,cr}_{20,nd}}-N_{{xy,cr}_{2,nd}}$ is almost constant. It can be proved that the values of the critical forces for $n_{d}=0$ derived in TM are identical to those for a homogeneous plate made of concrete with the appropriate value of the $E_{m}$ modulus.

**Figure 9:**
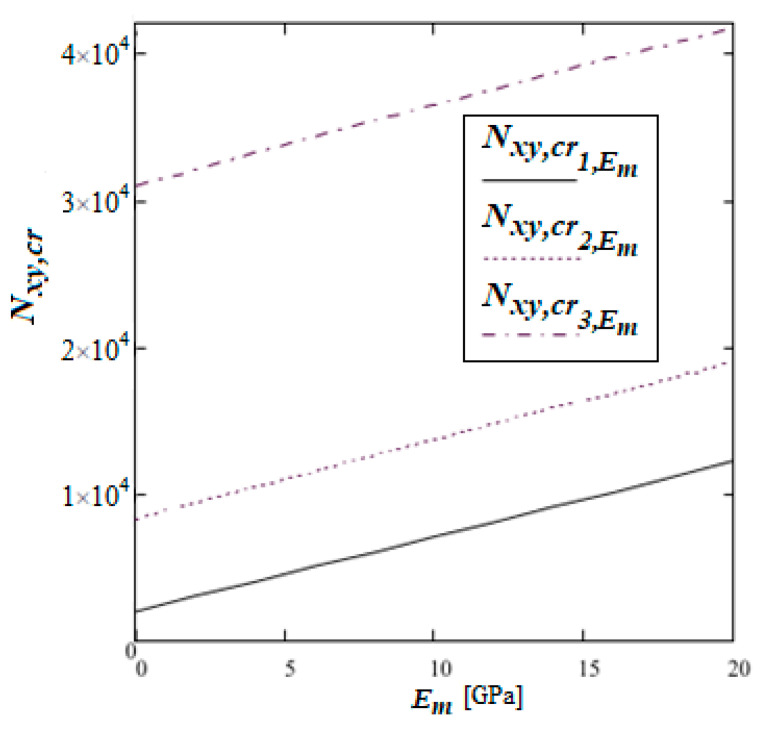
Critical shear forces evaluated for composites characterised by different $n_{d}$ (cf. Table 3) versus the modulus of elasticity of the matrix $E_{m}$.

The influence of the matrix material on the values of the critical forces is shown in Figure 9. The calculations were performed for three values of the proportion of the rib material in the cell: $n_{d}=0.025\left(N_{{xy,cr}_{1,Em}}\right);n_{d}=0.1\left(N_{{xy,cr}_{2,Em}}\right)\;\mathrm{and}n_{d}=0.3\left(N_{{xy,cr}_{3,Em}}\right)$. The matrix material varies from $E_{m}=0.001\;GPa$ to $E_{m}=20\;GPa$.

In Figure 9, the linear dependence of the critical shear force value on the matrix elasticity modulus is easily noticeable. This relation is different for different $n_{d}$. Table 3 shows explicit relations between the modulus of elasticity of the matrix, parameter $n_{d}$ and critical shear force values in considered cases.

**Figure 10:**
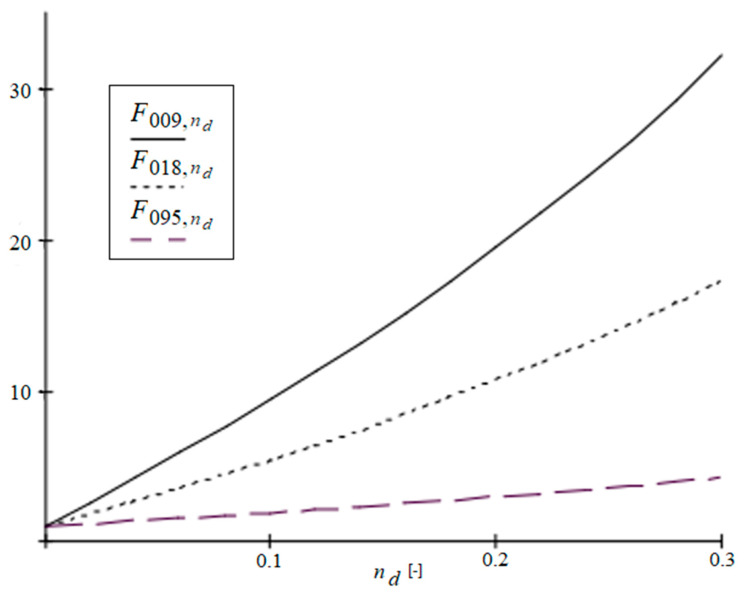
Functions $F_{009,nd},F_{018,nd},F_{095,nd}$ for different $B_{m}/B_{r}$ versus ratio $n_{d}\in \langle 0.00,0.30\rangle$.

Figure 10 presents the ratio of the critical force of a composite plate to the critical force of a homogeneous concrete plate $F_{B_{m}/B_{r},n_{d}}=N_{{xy,cr}_{comp}}/N_{{xy,cr}_{homog}}$ depending on the share of the rib material in the cell ($n_{d}$). These relations are evaluated for three ratios of the matrix stiffness to the rib stiffness $B_{m}/B_{r}$, which are obtained by altering elastic modulus of the matrix ($E_{m}=2\;GPa,4\;GPa,20\;GPa$). Functions: $F_{009,nd}$ for $B_{m}/B_{r}=0.009$, $F_{018,nd}$ for $B_{m}/B_{r}=0.018$ and $F_{095,nd}$ for $B_{m}/B_{r}=0.095$ are drawn in Figure 10.

It can be seen that for a smaller proportion of the matrix stiffness in the cell, an increase in the width of the ribs causes a faster increase in the value $N_{{xy,cr}_{comp}}/N_{{xy,cr}_{homog}}$. Such an observation is compliant with our engineering intuition, as the introduction of strong ribs into a relatively weak plate (low $B_{m}/B_{r}$ ratio) should result in a rapid increase in the critical force of the considered composite. For higher ratios of $B_{m}/B_{r}$ this reinforcement is not so meaningful, as the stronger matrix itself is capable of withstanding higher in-plane loadings without buckling.

## 4. Conclusions and Future Research

Let us present several of the most important conclusions which can be drawn from the presented work.

- The problem of stability in the thin plate with a dense system of microstructures is described by partial differential equations (PDE) with highly oscillating and discontinuous coefficients. Therefore, the tolerance averaging technique is applied to obtain averaged PDEs with functional but smooth coefficients.
- The obtained averaged PDEs have the same structure as the equations which describe the stability of orthotropic homogeneous plates. Hence, the solution of specific boundary problems of stability of certain composite plates can be obtained using simple mathematical methods. Such convenience in the modelling of complicated composites is superior to other known methods.
- The verification of the averaged equations of the tolerance model was carried out using the finite element method. The verification process of the model equations passed satisfactorily. In the case of plates with the share of rib material in the cell $n_{d}=d/l\leq 0.15$, the differences between the values of the critical forces obtained from both methods did not exceed 15%. Much more similar results of TM and FEM are obtained for lesser values of $n_{d}$. Consequently, it can be stated that the presented approach can be applied in multiple engineering issues.
- For plates which are simply supported on all edges, the relation between the shear critical force and the modulus of elasticity of the matrix $E_{m}$ is linear. This makes it possible to easily determine the value of the critical force for different values of $E_{m}$ without the need to solve differential equations. Consequently, the presented solution allows us to save a great deal of effort and computational resources.
- The influence of the rib width d on the critical shear force is particularly advantageous in plates with a low $B_{m}/B_{r}$ rate. In such a case, an introduction of a dense system of ribs allows us to significantly reinforce the whole composite. This is particularly important when the considered plates are expected to have good thermal insulation properties, which usually are reserved for materials with lesser density and poor elastic modulus $E_{m}$.

The presented analysis can be significantly expanded by multiple issues: the stability analysis of microperiodic plates situated on microheterogeneous foundations, the analysis of FGM plates with a dense system of ribs or plates having different shapes (e.g., circular). All of those issues can also be investigated with the use of experiments, which should be considered as the ultimate verification of the presented analysis.

## Figures and Tables

**Table 1:** The critical forces $P_{cr}$ of a biperiodic rectangular plate compressed in the $x_{1}$ direction obtained by tolerance averaging technique (TM) and FEM.

No.	*d* [m]	$n_{d}$ [-]	$P_{cr}$ [kN/m]		Relative Error
			TM	FEM	[(TM − FEM)/TM]
1	0.00	0	4284	4164	2.8%
2	0.005	0.025	5165	4847	6.2%
3	0.007	0.035	5525	5118	7.4%
4	0.01	0.05	6074	5529	9.0%
5	0.015	0.75	7016	6265	10.7%
6	0.02	0.1	7996	7034	12.0%
7	0.025	0.125	9019	7869	12.8%
8	0.03	0.15	10,089	8763	13.1%

**Table 2:** The critical shear forces $N_{xy,cr}$ of biperiodic rectangular plate obtained within tolerance averaging technique (TM) and FEM.

No.	*d* [m]	$n_{d}$ [-]	$N_{xy,cr}$ [kN/m]		Relative Error
			TM	FEM	[(TM − FEM)/TM]
1	0.00	0	10,002	9692	3.1%
2	0.005	0.025	12,188	11,317	7.1%
3	0.007	0.035	13,052	11,960	8.4%
4	0.01	0.05	14,366	12,937	9.9%
5	0.015	0.75	16,615	14,615	12.0%
6	0.02	0.1	18,950	16,503	12.9%
7	0.025	0.125	21,379	18,446	13.7%
8	0.03	0.15	23,913	20,518	14.2%

**Table 3:** The relationship between the critical shear force values and the matrix elasticity modulus for different $n_{d}=d/l$, where $E_{m}$ is the modulus of elasticity of the matrix.

No.	$n_{d}$ [-]	$N_{xy,cr}$ [kN/m]
1	0.025	$510\cdot E_{m}+2004$
2	0.1	$530\cdot E_{m}+5388$
3	0.3	$550\cdot E_{m}+30,938$

## Data Availability

The original contributions presented in this study are included in the article. Further inquiries can be directed to the corresponding author.

## Abbreviations

The following abbreviations are used in this manuscript:

FEM	Finite element method
FGM	Functionally graded materials
TM	Tolerance model
PDE	Partial differential equation
